# Publisher Correction: Global increase in tropical cyclone ocean surface waves

**DOI:** 10.1038/s41467-024-44909-9

**Published:** 2024-01-17

**Authors:** Jian Shi, Xiangbo Feng, Ralf Toumi, Chi Zhang, Kevin I. Hodges, Aifeng Tao, Wei Zhang, Jinhai Zheng

**Affiliations:** 1 Key Laboratory of Ministry of Education for Coastal Disaster and Protection, Hohai University, Nanjing, China; 2 College of Harbor, Coastal and Offshore Engineering, Hohai University, Nanjing, China; 3 National Centre for Atmospheric Science and Department of Meteorology, University of Reading, Reading, UK; 4 Department of Physics, Imperial College London, London, UK; 5 The National Key Laboratory of Water Disaster Prevention, Nanjing, China

**Keywords:** Ocean sciences, Ocean sciences

Correction to: *Nature Communications* 10.1038/s41467-023-43532-4, published online 03 January 2024

In the original PDF of the article, Figure 2 was truncated; the figure should have appeared as shown below
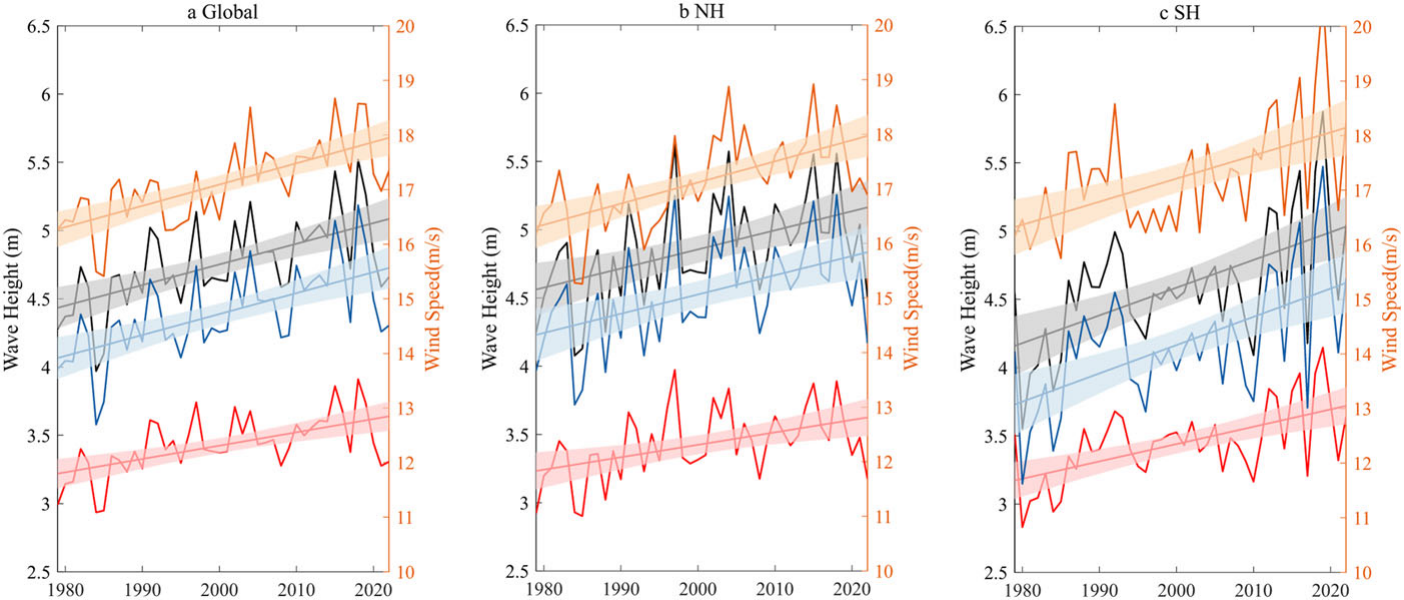


The original article PDF has been corrected. The HTML version was unaffected.

