# Supplementary material for: Global increase in tropical cyclone ocean surface waves
Source: Nat Commun. 2024 Jan 3;15:174. doi: 10.1038/s41467-023-43532-4 (PMC10764719; doi:10.1038/s41467-023-43532-4)
Supplement: Supplementary file 1 — Supplementary Information [file 41467_2023_43532_MOESM1_ESM.pdf]

Supplementary Information

Global increase in tropical cyclone ocean surface waves

Jian Shi[1,2], Xiangbo Feng[3,4], Ralf Toumi[4], Chi Zhang[2,5], Kevin I. Hodges[3], Aifeng Tao[1,2], Wei Zhang[2,5], Jinhai Zheng[1,2*]

1. Key Laboratory of Ministry of Education for Coastal Disaster and Protection, Hohai University, Nanjing, China
2. College of Harbor, Coastal and Offshore Engineering, Hohai University, Nanjing, China
3. National Centre for Atmospheric Science and Department of Meteorology, University of Reading, Reading, UK
4. Department of Physics, Imperial College London, London, UK.
5. The National Key Laboratory of Water Disaster Prevention, Nanjing, China

*Corresponding author Email: jhzheng@hhu.edu.cn

This PDF file includes

Supplementary Discussion

Supplementary Figures 1-16

Supplementary Tables 1-4

Supplementary Discussion

Climatology of TC waves

This section shows the climatology of tropical cyclone wave (TCW) metrics. First, we calculate the climatological mean of the maximum height of 6-hourly TCWs. The global average of the maximum height of 6-hourly TCWs is 4.8m. In individual ocean basins (Supplementary Fig. 1), the South Pacific (SP), South Indian Ocean (SI), North Atlantic (NA) and western North Pacific (WNP) see the largest values of the maximum height (4.4–5.3m), while the eastern North Pacific (EP) and North Indian Ocean (NI) have the smallest values (4.0m). The inter-basin variation in the climatology of the maximum height is related to the climatological TC intensity. The averaged 6-hourly TC intensity in the WNP, NA, SI and SP is larger (surface wind speed ≥16.8 m/s), contrasting with relatively weaker intensity in the NI and EP (surface wind speed ≤ 15.5m/s). The average height within the TCW footprint is much consistent with different ocean basins (2.9–3.5m).

Supplementary Fig. 1 also shows the climatology of TCW area and energy. Compared to the northern Hemisphere, the TCW area (i.e., the area of TC wave footprint) is larger in the southern Hemisphere with average value of 2.2×10^6 km^2, which is consistent with Fig. 1. In the northern Hemisphere, the largest area value of 1.4×10^6 km^2 is in the WNP, and the values in other basins are around $0.8\text{-}1.0\times10^6$ km^2. The TCW energy is an essential descriptor for long-term behaviour of TC waves. The TCW energy has large variations, with the biggest values in the WNP (9.2×10^{15} kJ) and SI (7.8×10^{15} kJ), due to both high TC frequency and large TCW area in the two basins. The TCW energy has the smallest values in the NI and EP, related to both small TC frequency and small TCW area. Thus, the climatology of the basin-wide TCW metrics is well determined by the measures of TC, including intensity, frequency, and length of lifetime.

Supplementary Figures

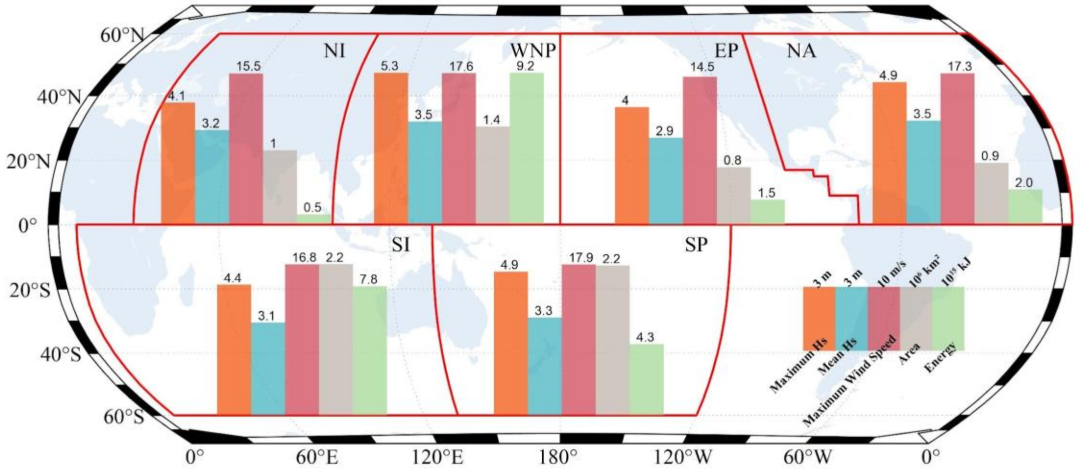

Supplementary Fig. 1 | Climatology of the maximum and mean height of TC wave footprint, the maximum winds of TCs, the area of TC wave footprint, and the TC wave energy in each basin in ERA5 over 1979-2022. ERA5 TC tracks are matched and truncated to IBTrACS. Wave height is represented by significant wave height (Hs). The maximum Hs is the 44-year mean of 6-hourly maximum height in the TC wave footprint. The mean Hs is the 44-year mean of 6-hourly mean height in the TC wave footprint. The maximum wind speed is the 44-year mean of 6-hourly 10m maximum wind speed. The TC wave area is the 44-year mean of 6-hourly area within the closed contour of Hs=2.5m. The TC wave energy is the 44-year mean of annually accumulated TC wave energy. The 44-year means of TC count for the WNP, EP, NA, NI, SI and SP basins are 21, 8, 8, 5, 13 and 9, respectively.

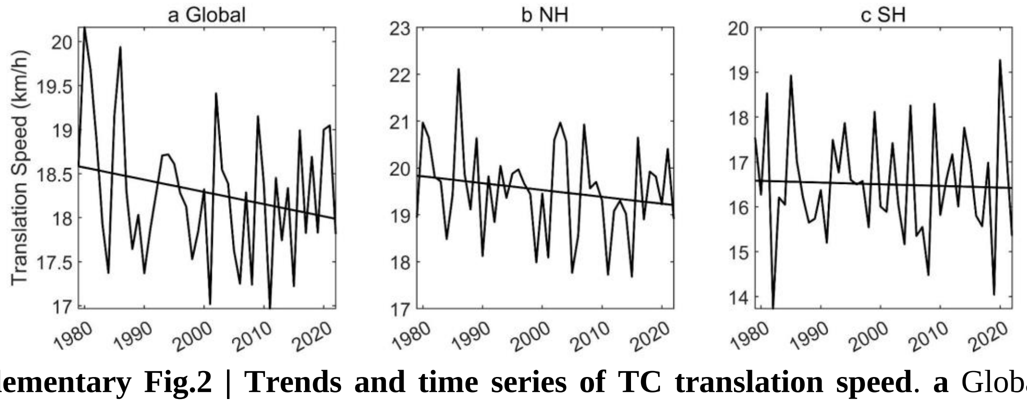

Supplementary Fig.2 | Trends and time series of TC translation speed. **a** Global trend (straight line) and time series (solid line) of annual mean TC translation speed (km/h) from ERA5 tracks over 1979–2022. **b-c** as **a**, but for the NH and SH. TC tracks are ERA5 tracks that are matched and truncated to IBTrACS. Note the straight lines without shading indicate that the trends do not pass the significance test.

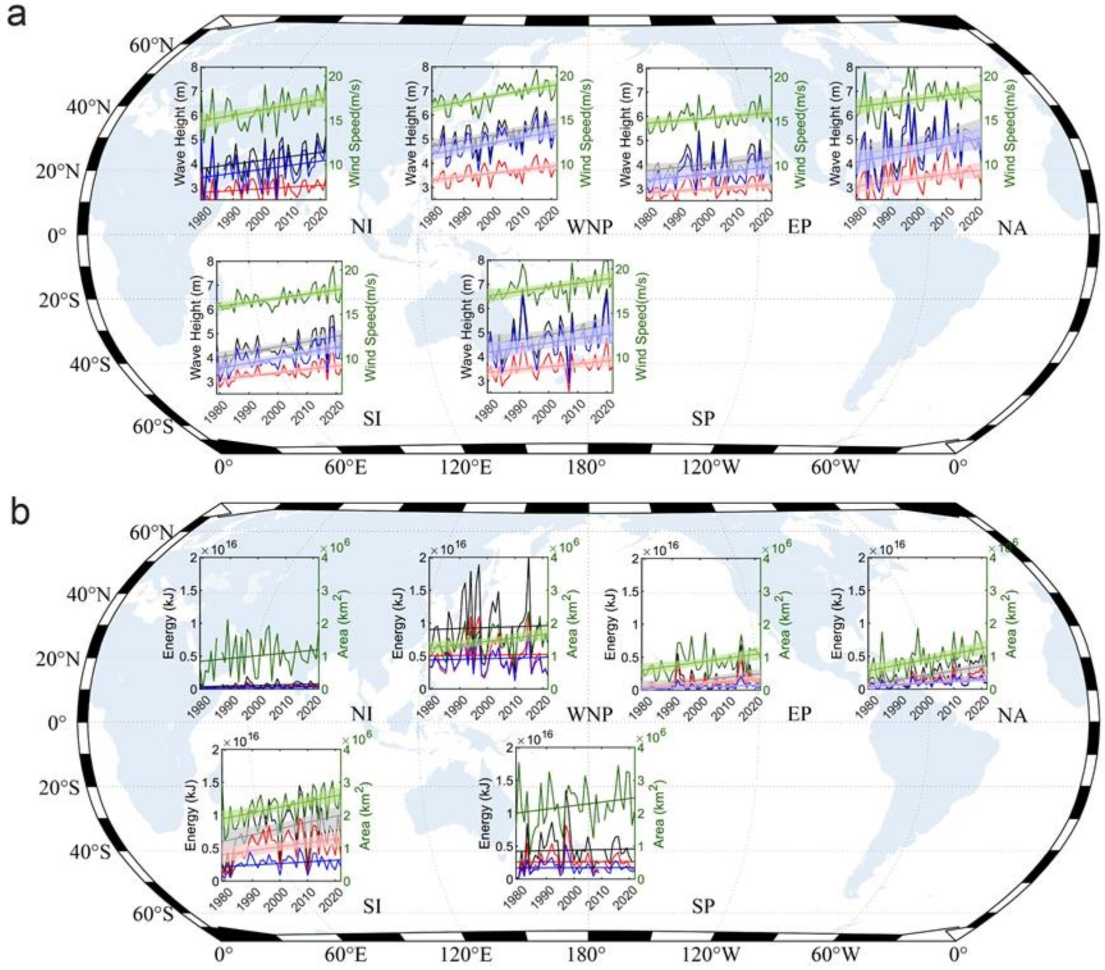

Supplementary Fig. 3 | Time series and trends of maximum height of the TC wave footprint, and TC wave energy, in each basin. **a** Basin-wide trends (straight line) and time series (solid line) of the annual mean of six-hourly maximum height for mixed (black), swell (red) and wind (blue) waves over 1979–2022. Annual 6-hourly 10m maximum wind speed of TCs (green) is also provided in **a**. **b** Basin-wide trends (straight line) and time series (solid line) of annually accumulated TC wave energy for mixed (black), swell (red) and wind-sea (blue) waves over 1979-2022. Annual mean TC wave area (green) is also provided in **b**. TC tracks are ERA5 tracks that are matched and truncated to IBTrACS. Shading shows the 95% confidence interval for the significant trend; straight lines without shading indicate that the trends do not pass the significance test at the 95% level, but many of them are significant at the 90% level, see Supplementary Table 1. Wave height is represented by significant wave height (Hs). Maximum height is defined by the maximum value of Hs within the 6-hourly TC wave footprint; the annual 6-hourly maximum height is the maximum height averaged over each year.

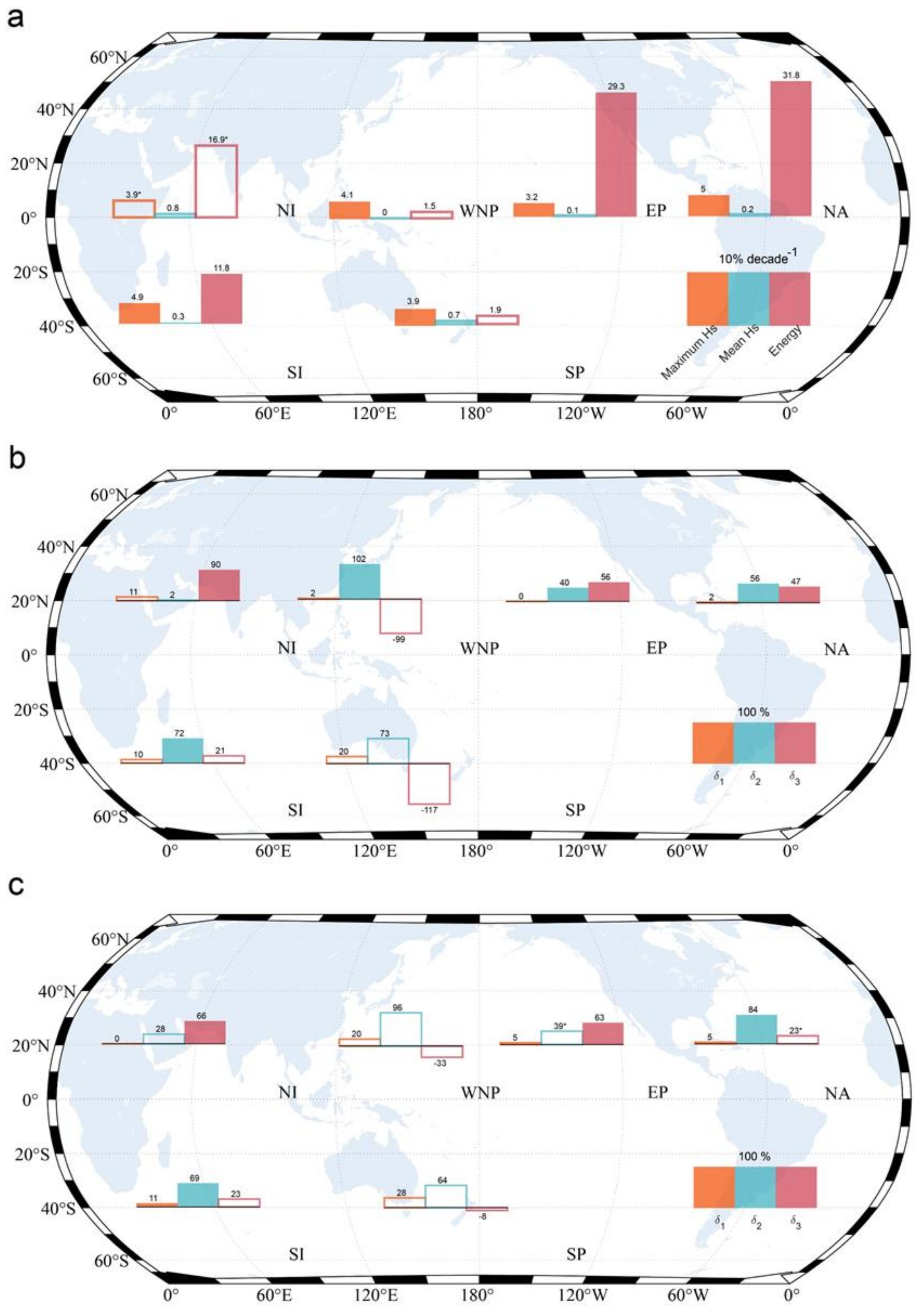

Supplementary Fig. 4 | Trend values of the height of TC waves, and trends of decompositions of TC wave energy. a Basin-wide trend values of the annual mean six-hourly maximum height (orange bar; %/decade), mean height (blue bar; %/decade), and annually accumulated energy of TC waves (red bar; %/decade), derived from ERA5 over 1979–2022. **b** Contribution (%) of linear effects (first-order terms) of the height (orange bar), area (blue bar)

and annual accumulated duration (red bar) of TC waves to the trend in annual TC wave energy, derived from ERA5 over 1979–2022. In **a** and **b**, TC tracks are ERA5 tracks that are matched and truncated to IBTrACS. **c** as **b**, but for the full ERA5 tracks that are matched to IBTrACS (T2 in Supplementary Tables 1 and 2). Filled and unfilled boxes represent significant and insignificant trends at the 95% confidence level, respectively, while * indicates the trends that are significant at the 90% confidence level. Wave height is represented by significant wave height (Hs).

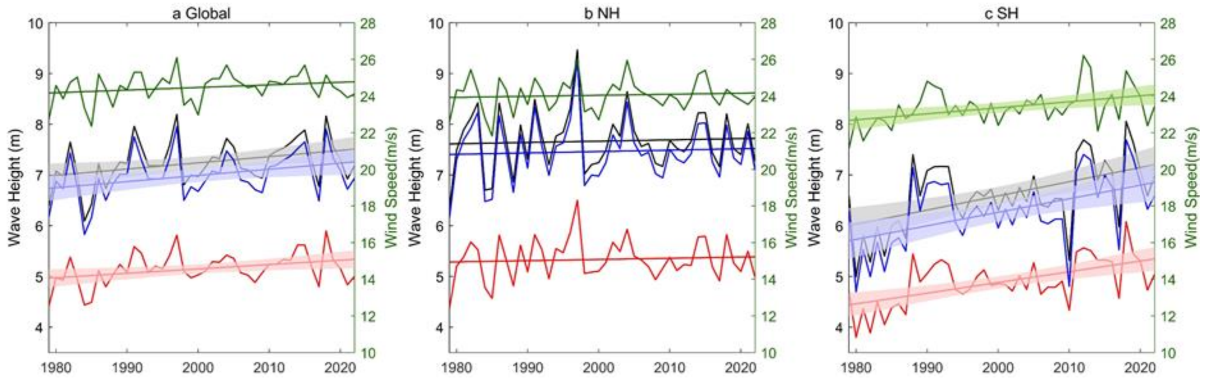

Supplementary Fig. 5 | Trends and time series of lifetime-maximum height of TC waves. **a** Global trend (straight line) and time series (solid line) of the annual mean of lifetime-maximum height of TC waves for mixed (black), swell (red) and wind (blue) sea waves in ERA5 over 1979–2022. The annual mean of TC lifetime-maximum 10m wind speed (green) is also provided in **a**. **b-c** as **a**, but for the NH and SH. TC tracks are ERA5 tracks that are matched and truncated to IBTrACS. Shading shows the 95% confidence interval for the significant trend; straight lines without shading indicate that the trends do not pass the significance test. Wave height is represented by significant wave height (Hs).

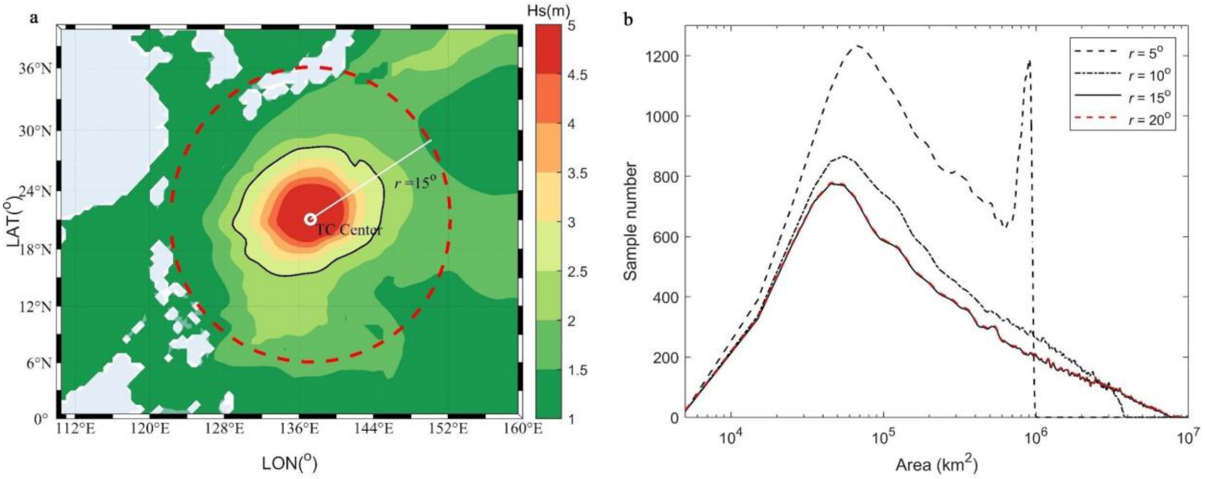

Supplementary Fig. 6 | Calculation TC wave footprint and sample size distribution of TC wave area in ERA5. a Spatial distribution of the 6-hourly height of TC waves at 2018-09-01 12 UTC during TC Jebi (2018) in the Western North Pacific. Wave height is represented by significant wave height (Hs). The red dashed circle represents the area with a 15° geodesic

radius in the first guess of TC waves. Contours show the values of Hs, with a black line for the continuous area where Hs≥2.5 m; this continuous area is finally defined as the TC wave area. **b** Sample size distribution of 6-hourly TC wave area with radius of 5°, 10°, 15° and 20° in the first guess.

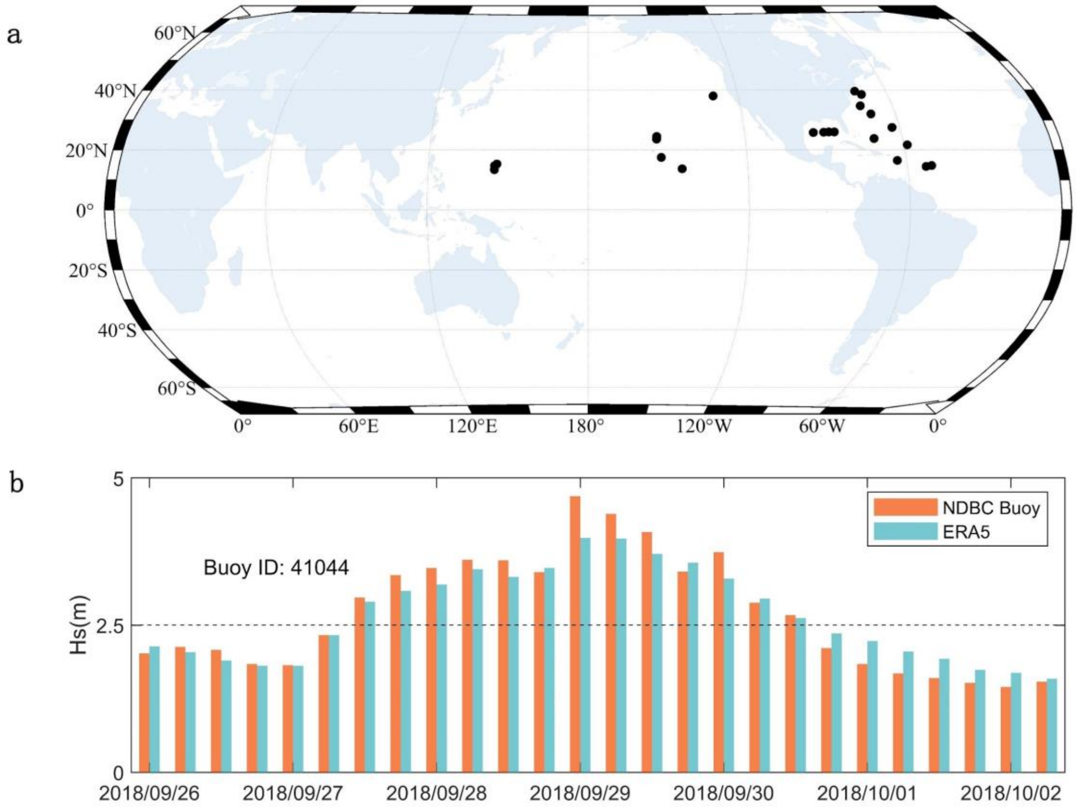

Supplementary Fig. 7 | Buoy locations and schematic diagram of calculating the TC wave duration. a Locations of 22 NDBC buoys used in the validation of TC wave duration for individual TCs. **b** Demonstration diagram of calculating the TC wave duration for TC Kirk (2018). The TC wave duration for Hs ≥2.5m is calculated for both the buoy (ID:41044) and ERA5, when the buoy is within the 15-degree geodesic circle during the lifetime of TC Kirk. In the case of TC Kirk, the number of 6-hourly time steps with TC wave Hs ≥2.5m represents the TC wave duration. Time steps above the black dashed line are 13; so the TC wave duration is 3.25 days for the buoy and ERA5.

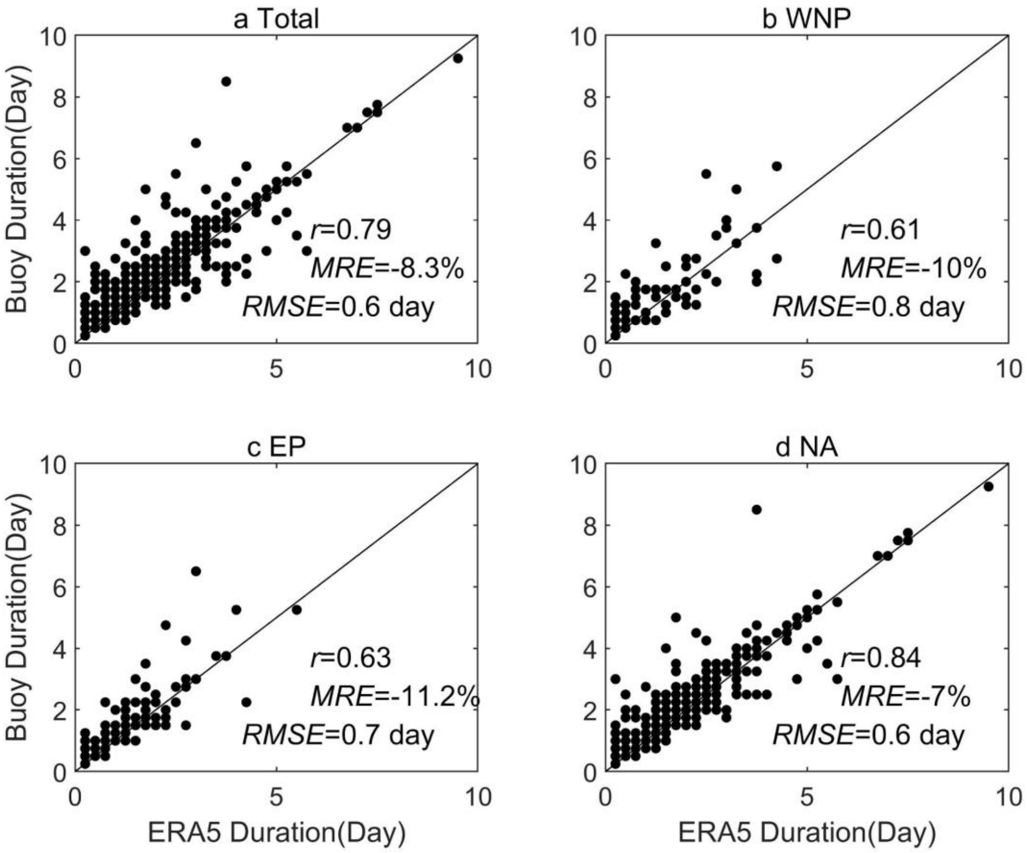

Supplementary Fig. 8 | Comparison of TC wave duration between NDBC buoy observation and ERA5 reanalysis. Twenty two buoys during 1979–2018 are used, with 582 TC events identified in the validation. **a** Scatter plot of TC wave duration for the 582 TC events, **b-d** scatter plots for TC events in the WNP, EP and NA, respectively. *r*, *MRE*, and *RMSE* represent Pearson correlation coefficient, mean relative error (relative to the buoy observation), and root-mean-square error, respectively. The number of TC events for the WNP, EP and NA is 72, 84 and 426, respectively.

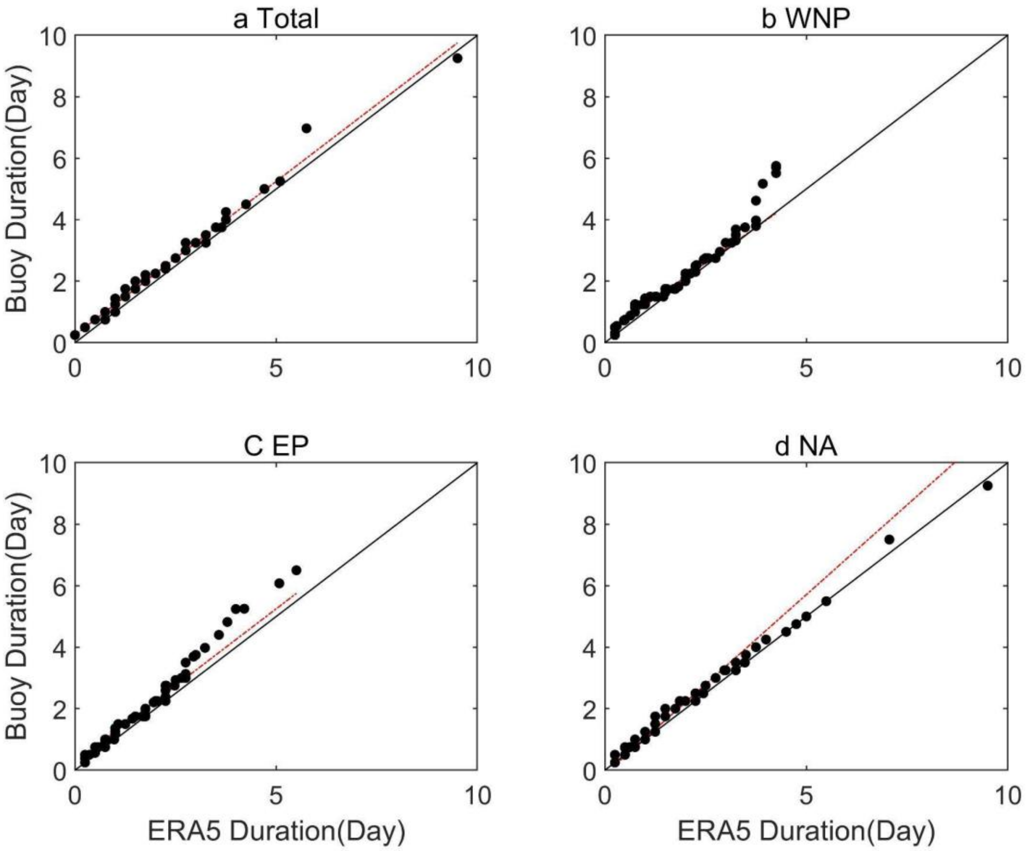

Supplementary Fig. 9 | Quantile-quantile (Q-Q) plot of TC wave duration between NDBC buoy observation and ERA5 reanalysis. Twenty two buoys during 1979–2018 are used, with 582 TCs identified in the validation. **a** Q-Q plot of TC wave duration for the 582 TC events, **b-d** Q-Q plots for TC events in the WNP, EP and NA, respectively. The red and black lines represent the quartile line and the 45-degree reference line. The number of TC events for the WNP, EP and NA is 72, 84 and 426, respectively.

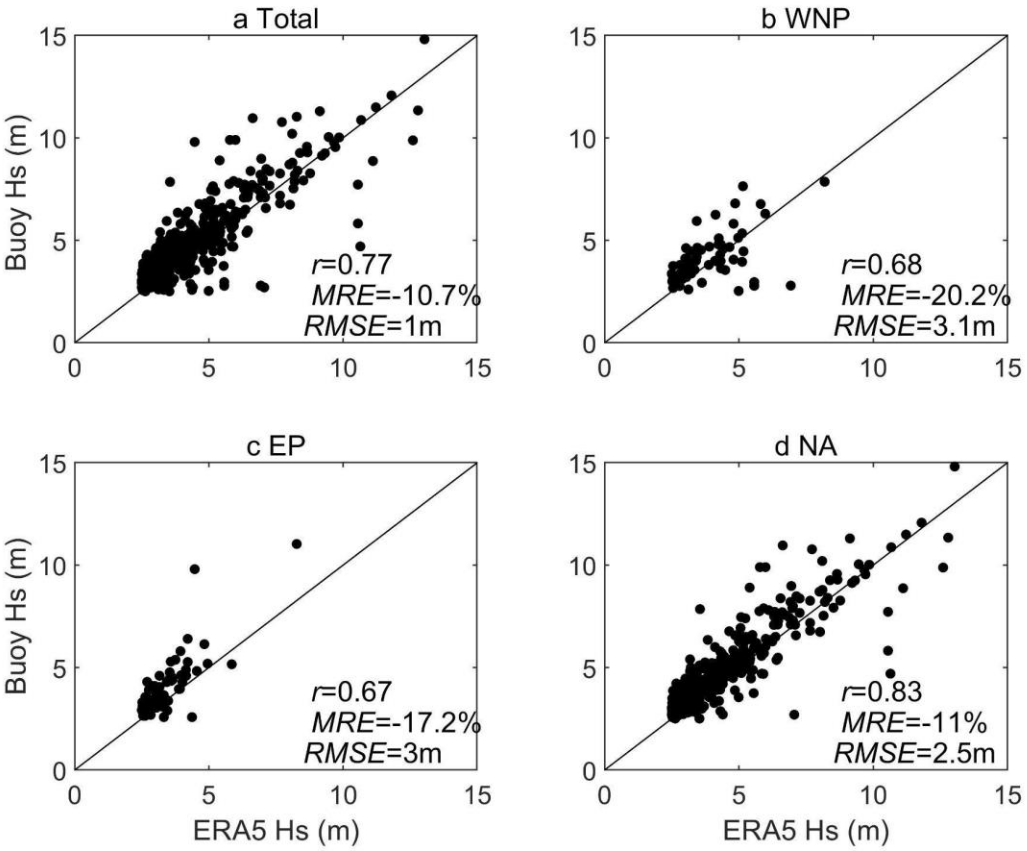

Supplementary Fig. 10 | Comparison of maximum height of TC waves between NDBC buoy observation and ERA5 reanalysis. Twenty two buoys during 1979–2018 are used, with 582 TC events identified in the validation. The dot represents the maximum wave height in the TC wave duration in observations and ERA5. **a** Scatter plot of maximum wave height for the 582 TC events, **b-d** scatter plots for TC events in the WNP, EP and NA, respectively. *r*, *MRE*, and *RMSE* represent Pearson correlation coefficient, mean relative error (relative to the IBTrACS data), and root-mean-square error, respectively. The number of TC events for the WNP, EP and NA is 72, 84 and 426, respectively.

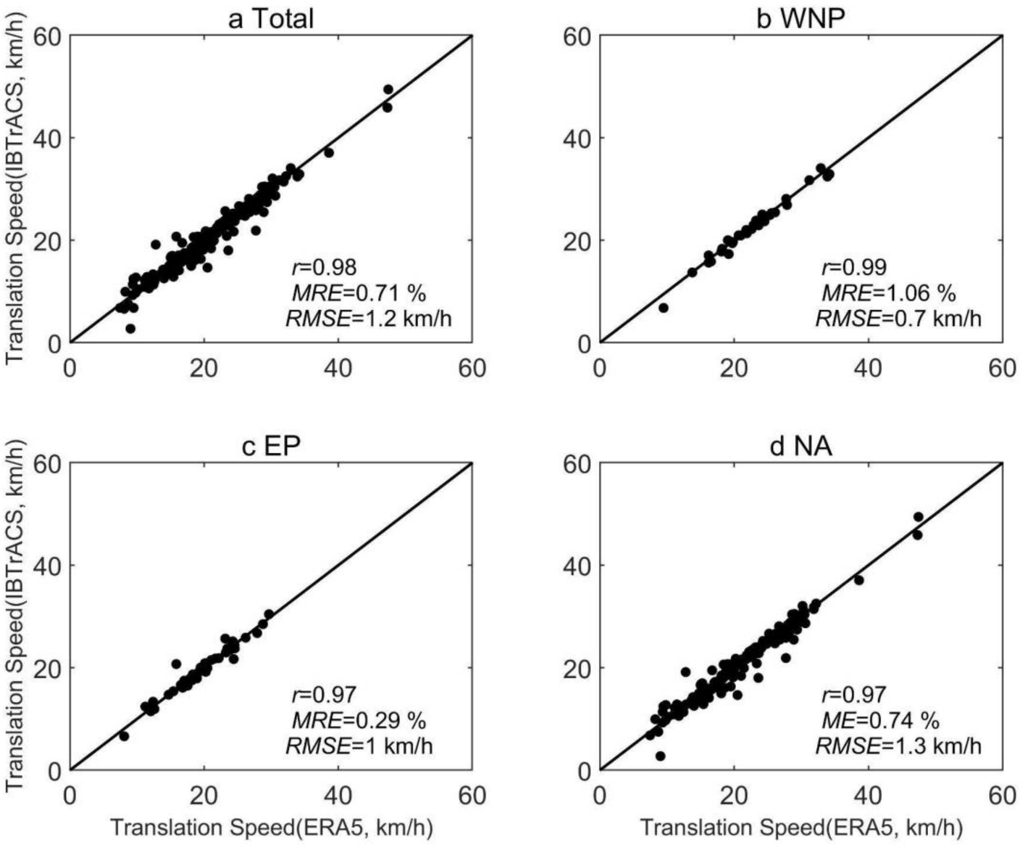

Supplementary Fig. 11 | Comparison of TC translation speed between IBTrACS and ERA5 reanalysis. a Scatter plot of TC translation speed for TCs used in the Supplementary Fig. 8, **b-d** scatter plots for TC events in the WNP, EP and NA, respectively. *r*, *MRE*, and *RMSE* represent Pearson correlation coefficient, mean relative error (relative to the IBTrACS data), and root-mean-square error, respectively. The number of TC events for the WNP, EP and NA is 72, 84 and 426, respectively.

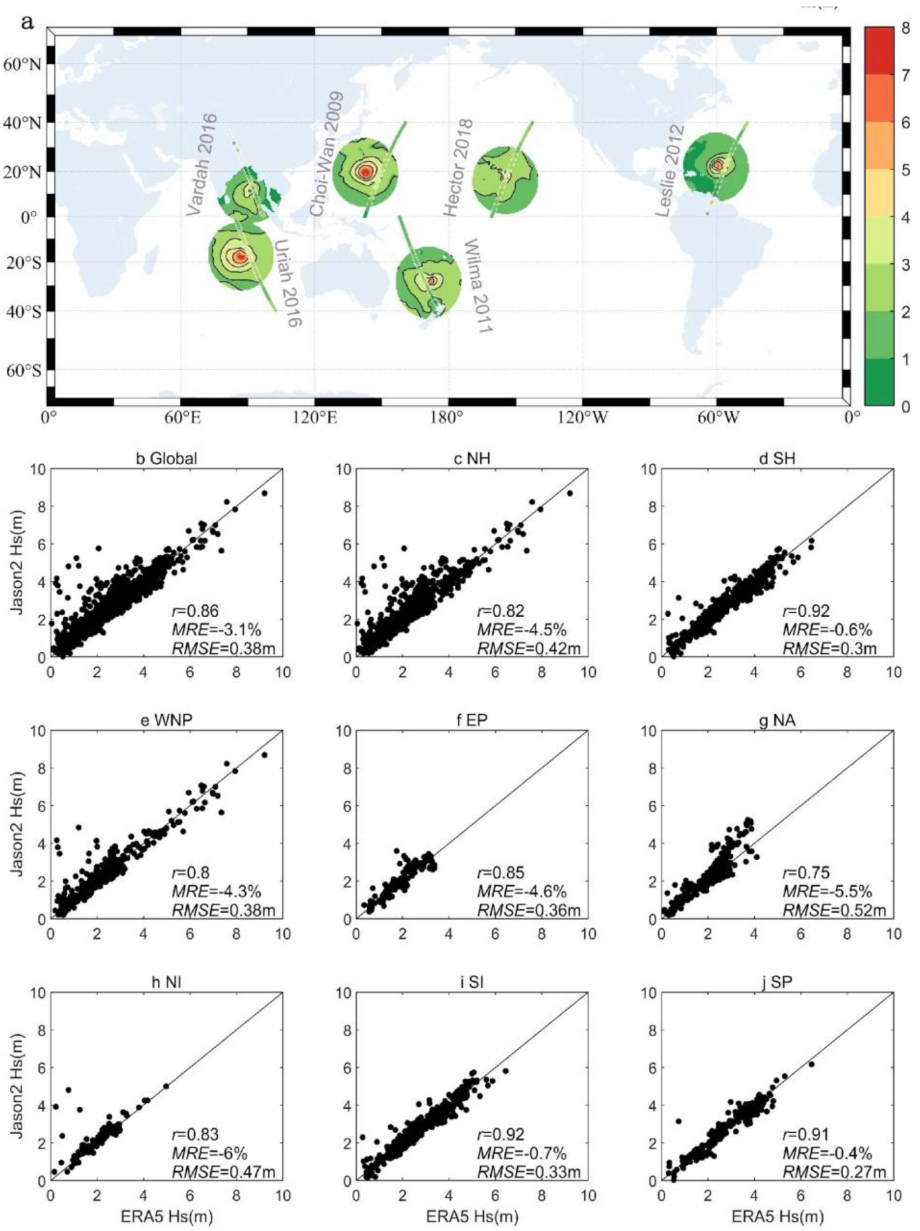

Supplementary Fig. 12 | Comparison of height of TC waves between Jason-2 satellite altimeter observations and ERA5 reanalysis from July 2008 to December 2018. a Demonstration of Jason-2 along-track significant wave height (Hs) and ERA5 Hs within a 15-degree geodesic circle centred at the TC position based on TC tracks in ERA5. Jason-2 along-track Hs (about 3-4 km track width, displayed here only for visual clarity) within the TC wave area are binned into the ERA5 grid (0.5×0.5 deg). Comparison is only for the times of TC lifetime-maximum intensity. **b-d** Scatter plots of ERA5 Hs vs. Jason-2 Hs for global and hemispheric TCs. A total of 1373 samples are finally found in 51 TC events in this validation. The number of TCs for the WNP, EP, NA, NI, SI and SP is 15, 5, 11, 5, 10 and 5, respectively. *r*, *MRE* and *RMSE* represent Pearson correlation coefficient, mean relative error, and root-mean-square error, respectively.

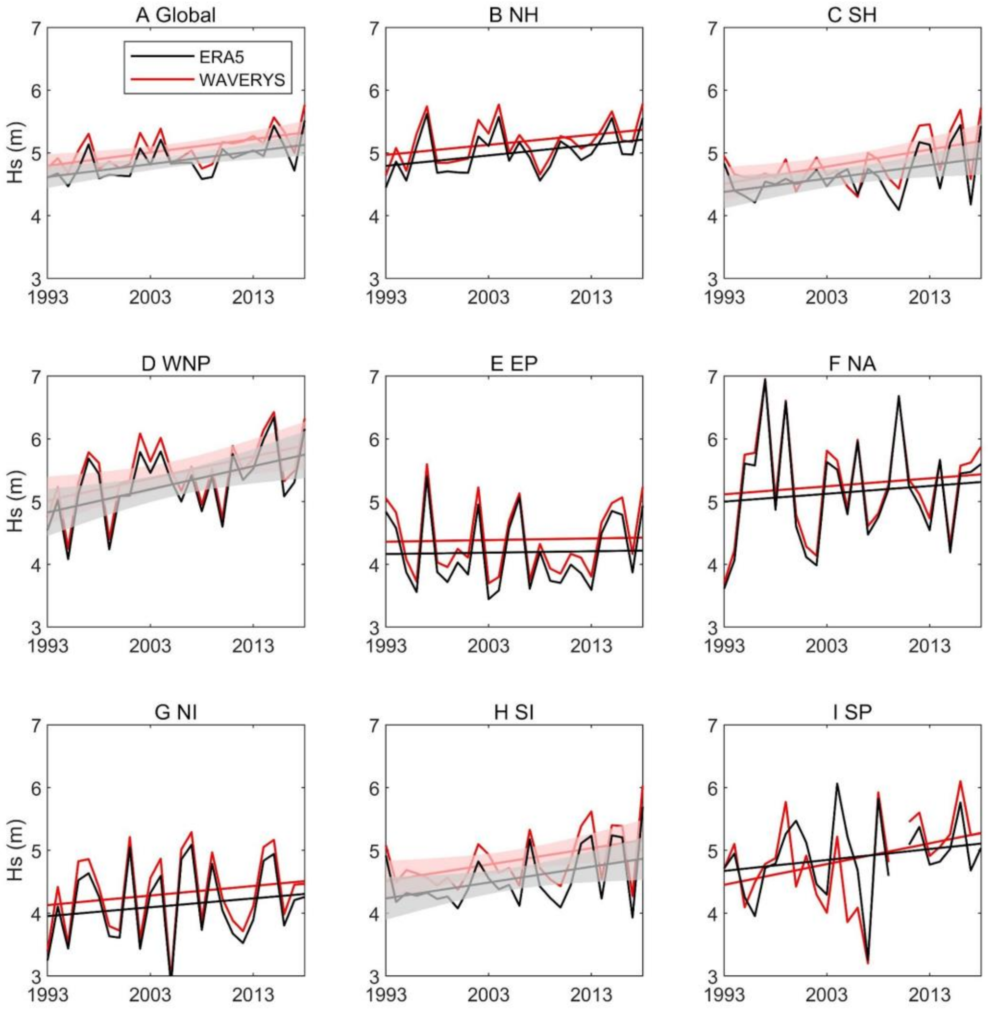

Supplementary Fig. 13 | Global and basin-wide trends and time series of the maximum height of TC wave footprint. a-c Global, NH and SH trends (straight line) and time series (solid line) of the annual mean of 6-hourly maximum height of TC waves for mixed waves in ERA5 (black) and WAVERYS (red) over 1993–2018. **d-i** as **a**, but for basin-wide values. Shading shows the 95% confidence interval for the significant trend; straight lines without shading indicate that the trends do not pass the significance test at the 95% level.

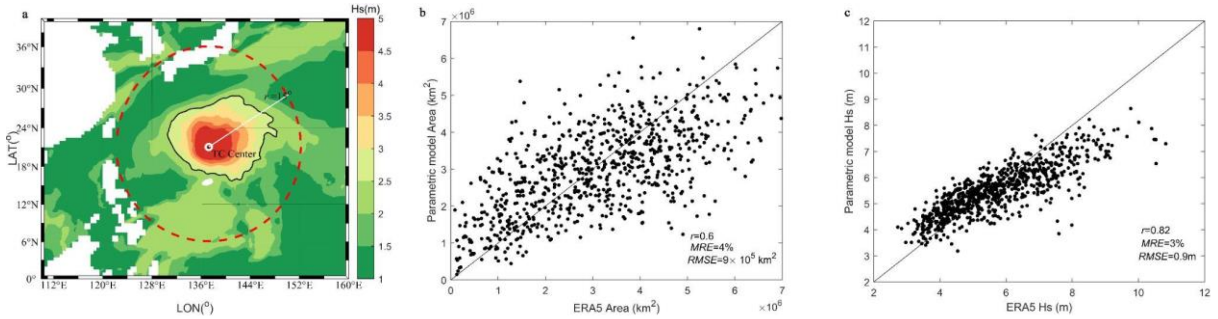

Supplementary Fig. 14 | Comparison of TC wave footprint, TC wave area and maximum TC wave height against parametric wave model. a Spatial distribution of the 6-hourly height of TC waves produced by the parametric wave model[63], at 2018-09-01 12 UTC during TC Jebi (2018) in the Western North Pacific, corresponding to the wave field in ERA5 in Supplementary Fig.6a. The red dashed circle represents the area with a 15° geodesic radius in the first guess of TC waves. Contours show the values of Hs, with a black line for the continuous area where Hs≥2.5 m. **b** Scatter plot of the average of 6-hourly TCW area over the TC lifetime based on the parametric wave model and ERA5, for the 809 TCs. **c** Scatter plot of the average of 6-hourly maximum height over the TC lifetime based on the parametric wave model and ERA5, for the 809 TCs. Note that the parametric wave model is valid only for the time when storm intensity is restricted to 30m/s < V_{max} < 70m/s (see "Comparison to parametric wave model " in subsection 5 of the Methods section). In b-c, only the common time when both datasets have valid values is considered.

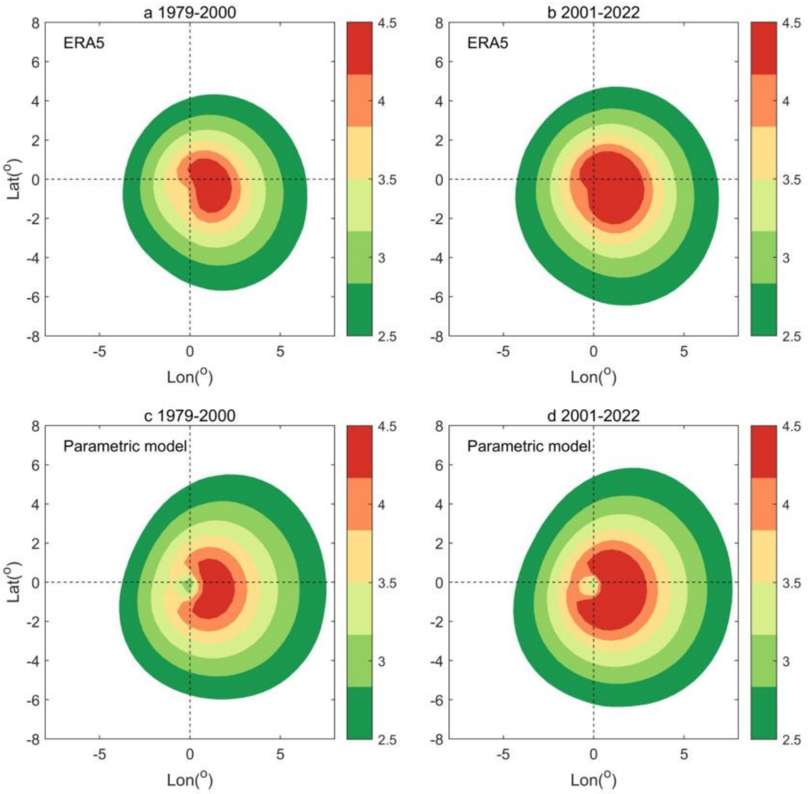

Supplementary Fig. 15 | Composite mean of the height of TC wave footprint in the NH, from ERA5 and from parametric wave model. a-b Composite mean of the 6-hourly wave height (m) around the TC track position in ERA5, for the two epochs 1979–2000 and 2001–2022. **c-d** as a-b, but i from the parametric wave model[63]. Note that the parametric wave model is valid only for the time when storm intensity is restricted to 30m/s < V_{max} < 70m/s (see "Comparison to parametric wave model " in subsection 5 of the Methods section). Only the common time when both datasets have valid values is considered. Thus, the samples of TC wave footprint in a-b are different from those in Fig.1a-b, which include all the footprints in the time periods.

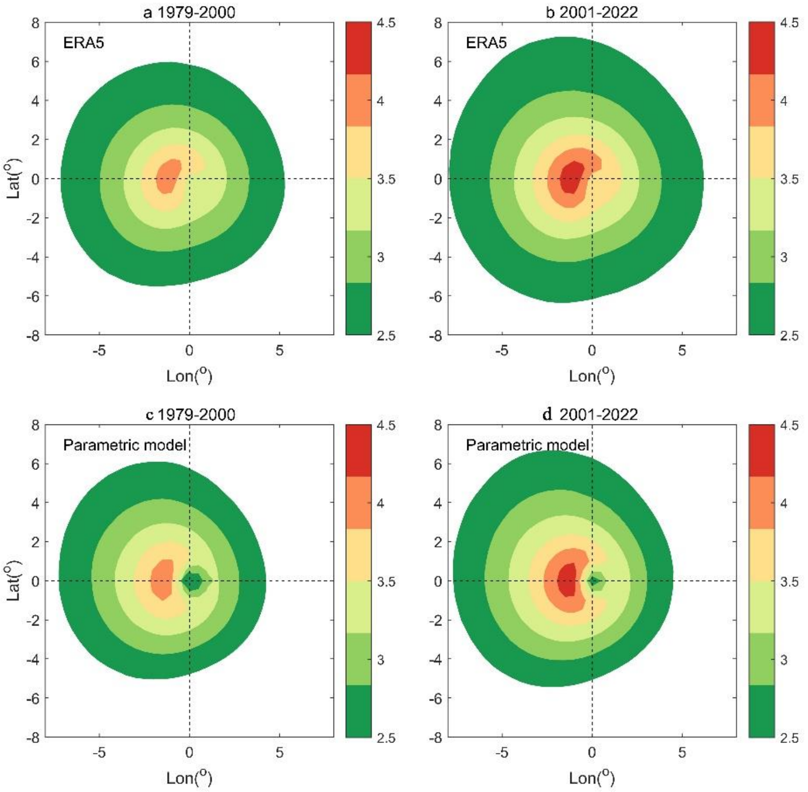

Supplementary Fig. 16 | Composite mean of the height of TC wave footprint in the SH. a-b Composite mean of the 6-hourly wave height (m) around the TC track position in ERA5, for the two epochs 1979–2000 and 2001–2022. **c-d** as a-b, but in a parametric wave model[63]. Note that the parametric wave model is valid only for the time when storm intensity is restricted to 30m/s < V_{max} < 70m/s (see "Comparison to parametric wave model " in subsection 5 of the Methods section). Only the common time when both datasets have valid values is considered. Thus, the samples of TC wave footprint in a-b are different from those in Fig.1d-e, which include all the footprints in the time periods.

Supplementary Tables

Supplementary Table 1 | Linear trends in TC intensity (maximum sustained wind) and height of TC wave footprint, at global and basin-wide scales, based on two types of TC datasets from ERA5 over 1979–2022. Relative trends are also provided, which are the absolute trend values relative to the 44-year mean. Types of TC track include: the ERA5 tracks that are matched and truncated to IBTrACS (T1), and the full ERA5 tracks that are matched to IBTrACS (T2). The last two lines are linear trends of ERA5 and WAVERYS reanalysis over 1993-2018; the relative values are relative to the mean over 1993–2018. * indicates a significant trend at the 95% confidence level. ** indicates a significant trend at the 90% confidence level. Trends without * or ** do not pass the significance test at the 90% level.

			Global	NH	SH	WNP	EP	NA	NI	SI	SP
ERA5 TC tracks (T1) (1979–2022)	TC intensity	6-hourly-Max wind speed m/s /decade(%)	0.39±0.13* (2.3±0.8*)	0.39±0.15* (2.3±0.9*)	0.42±0.20* (2.5±1.2*)	0.60±0.24* (3.4±1.4*)	0.28±0.25* (1.8±1.6*)	0.42±0.38* (2.4±2.2*)	0.59±0.40* (3.6±2.5*)	0.46±0.23* (2.8±1.4*)	0.47±0.31* (2.6±1.8*)
		Lifetime-Max wind speed m/s /decade(%)	0.14±0.18 (0.6±0.7)	0.06±0.21 (0.2±0.9)	0.33±0.22* (1.4±0.9*)	0.01±0.35 (0.4±1.4)	0.25±0.36 (1.2±1.7)	0.63±0.51* (2.6±2.1*)	0.45±0.55 (2.1±2.5)	0.33±0.28* (1.4±1.2*)	0.32±0.49 (1.4±2.1)
	Mixed wave	6-hourly-Max Hs m/decade(%)	0.15±0.06* (3.2±1.3*)	0.14±0.08* (2.9±1.6*)	0.20±0.09* (4.4±1.9*)	0.21±0.11* (4.1±2.1*)	0.13±0.12* (3.2±3.0*)	0.24±0.20* (5.0±4.2*)	0.16±0.17** (3.9±4.1**)	0.22±0.10* (4.9±2.3*)	0.19±0.17* (3.9±3.4*)
		Lifetime-Max Hs m/decade(%)	0.12±0.10* (1.7±1.4*)	0.03±0.14 (0.3±1.8)	0.28±0.14* (4.2±2.1*)	0.01±0.23 (0.2±2.7)	0.12±0.20 (2.0±3.5)	0.31±0.35 (4.1±4.7)	0.19±0.24 (3.3±4.2)	0.34±0.16* (5.4±2.6*)	0.23±0.28 (3.2±4.0)
		6-hourly-Max Hs (depth>100m)m/decade(%)	0.15±0.06* (3.3±1.2*)	0.14±0.08* (2.9±1.6*)	0.22±0.09* (4.7±1.9*)	0.22±0.11* (4.3±2.1*)	0.13±0.12* (3.3±3.0*)	0.25±0.20* (5.1±4.2*)	0.16±0.17** (4.0±4.1**)	0.23±0.10* (5.2±2.3*)	0.21±0.17* (4.1±3.3)
	Swell	6-hourly-Max Hs m/decade(%)	0.10±0.04* (2.8±1.2*)	0.09±0.05* (2.7±1.6*)	0.13±0.05* (3.7±1.6*)	0.13±0.07* (3.7±2.0*)	0.09±0.08* (2.6±2.5*)	0.16±0.14* (4.6±4.0*)	0.07±0.09** (2.5±3.2**)	0.14±0.06* (4.1±1.9*)	0.11±0.10* (3.2±2.9*)
	Wind sea	6-hourly-Max Hs m/decade(%)	0.15±0.06* (3.5±1.4*)	0.14±0.08* (3.1±1.7*)	0.21±0.09* (5.0±2.1*)	0.22±0.11* (4.6±2.3*)	0.13±0.12* (3.7±3.5*)	0.23±0.20* (5.1±4.5*)	0.17±0.17** (4.4±4.6**)	0.22±0.10* (5.5±2.6*)	0.19±0.17* (4.19±3.8*)
ERA5 TC tracks (T2) (1979–2022)	Mixed wave	6-hourly-Max Hs m/decade(%)	0.09±0.05* (2.1±1.2*)	0.09±0.07* (2.3±1.7*)	0.10±0.08* (2.0±1.8*)	0.11±0.09* (2.6±2.1*)	0.09±0.09* (2.5±2.4*)	0.28±0.13* (7.2±3.4*)	0.12±0.11* (3.6±3.4*)	0.14±0.08* (3.4±2.0*)	0.06±0.12 (1.3±2.9)
WAVERYS (1993–2018)	Mixed wave	6-hourly-Max Hs m/decade(%)	0.23±0.13* (4.6±2.5*)	0.16±0.17** (3.1±3.3**)	0.30±0.19* (6.3±4.1*)	0.35±0.26* (6.4±4.8*)	0.03±0.32 (0.6±7.2)	0.13±0.45 (2.4±8.5)	0.15±0.35 (3.5±8.1)	0.25±0.23* (5.4±4.9*)	0.33±0.35 (6.8±7.2)
ERA5 TC tracks (T1) (1993–2018)	Mixed wave	6-hourly-Max Hs m/decade(%)	0.20±0.12* (4.2±2.5*)	0.17±0.17** (3.3±3.4**)	0.21±0.18* (4.6±3.9*)	0.37±0.26* (7.0±4.9*)	0.02±0.32 (0.5±7.5)	0.12±0.47 (2.4±9.0)	0.14±0.33 (3.4±8.0)	0.25±0.23* (5.5±5.1*)	0.17±0.33 (3.6±6.7)

Supplementary Table 2 | Linear trends in TC wave area, TC wave accumulated duration and TC wave energy, at global and basin-wide scales, based on two types of TC datasets from ERA5 over 1979–2022. Relative trends are also provided, which are the absolute trend values relative to the 44-year mean. Types of TC track include: the ERA5 tracks that are matched and truncated to IBTrACS (T1), and the full ERA5 tracks that are matched to IBTrACS (T2). * indicates a significant trend at the 95% confidence level. ** indicates a significant trend at the 90% confidence level. Trends without * or ** do not pass the significance test at the 90% level.

			Global	NH	SH	WNP	EP	NA	NI	SI	SP
ERA5 TC tracks (T1) (1979–2022)	TC wave area	≥2.5m $10^5 km^2$ /decade(%)	0.91±0.61* (5.7±3.8*)	0.70±0.64* (5.7±5.2*)	1.93±0.82* (8.7±3.7*)	1.01±0.83* (6.9±5.6*)	1.18±0.84* (13.7±9.8*)	1.67±0.94* (18.3±10.2*)	0.13±1.56 (1.2±14.2)	1.71±0.98* (7.6±4.4*)	1.08±1.55 (4.8±6.9)
		≥3.5m $10^5 km^2$ /decade(%)	0.23±0.20* (5.4±4.7*)	0.09±0.23 (2.5±6.4)	0.65±0.31* (13.0±6.1*)	0.32±0.30* (6.7±6.3*)	0.13±0.17 (12.6±16.6)	0.49±0.38* (17.3±13.3*)	0.19±0.71 (6.1±22.5)	0.56±0.44* (11.8±9.2*)	0.56±0.67 (9.5±11.3)
		≥4.5m $10^5 km^2$ /decade(%)	0.07±0.08** (5.5±5.7**)	0.04±0.09 (3.1±7.1	0.19±0.11* (15.5±8.9*)	0.15±0.15** (8.0±8.1**)	0.04±0.06 (16.6±24.0)	0.18±0.16* (17.0±15.0*)	0.11±0.21 (15.9±28.5)	0.17±0.14* (16.8±14.5*)	0.19±0.28 (10.6±15.0)
		≥2.5m (depth>100m) $10^5 km^2$ /decade(%)	0.89±0.60* (5.7±3.9*)	0.68±0.64* (5.7±5.4*)	1.55±0.92* (6.9±4.1*)	0.94±0.83* (6.6±5.9*)	1.18±0.84* (13.7±9.8*)	1.67±0.93* (18.6±9.7*)	0.13±1.51 (1.2±14.2)	1.69±0.98* (7.5±4.4*)	1.04±1.55 (4.7±6.9)
	TC wave duration	Mixed wave day/decade(%)	11.9±26.6 (2.1±4.8)	15.2±14.2* (4.3±4.1*)	-3.4±17.2 (-1.7±8.4)	-16.0±12.1* (-8.4±6.3*)	13.9±9.1* (20.1±13.2*)	11.8±7.5* (17.0±10.9*)	3.1±2.1* (15.9±10.4*)	0.90±11.9 0.7±8.7)	-4.3±8.5 (-6.4±12.7)
	TC wave energy	Mixed wave 10^{15}kJ /decade(%)	2.31±1. 87* (8.9±7.3*)	1.36±1.20* (10.1±8.8*)	0.95±1.13** (7.8±9.2**)	0.15±1.00 (1.6±10.7)	0.42±0.33* (28.0±21.7*)	0.62±0.30* (29.3±14.4*)	0.09±0.10** (16.9±18.2**)	0.93±0.76* (11.8±9.7*)	0.08±0.68 (1.9±15.6)
		Swell 10^{15}kJ /decade(%)	1.54±1.30* (10.0±8.5*)	0.86±0.79* (11.4±10.5*)	0.67±0.54* (8.6±6.9*)	0.08±0.66 (1.5±13.2)	0.31±0.24* (30.3±23.7*)	0.41±0.18* (34.7±15.6*)	0.06±0.06* (19.5±19.0*)	0.66±0.51* (12.6±9.8*)	0.05±0.42 (2.1±16.0)
		Wind sea 10^{15}kJ /decade(%)	0.72±0.70* (6.9±6.6*)	0.48±0.43* (8.0±7.2*)	0.28±0.30** (6.3±6.8**)	0.06±0.40 (1.4±9.4)	0.13±0.11* (27.1±21.3*)	0.26±0.13* (28.1±13.9*)	0.04±0.05 (14.0±17.8)	0.28±0.26* (10.2±9.6*)	0.03±0.260 (1.2±14.9)
ERA5 TC tracks (T2) (1979–2022)	TC wave energy	Mixed wave 10^{15}kJ /decade(%)	3.11±2.80* (8.8±7.8*)	2.01±1.57* (11.6±9.1*)	1.09±1.78 (6.1±10.0)	0.45±1.25 (4.1±11.4)	0.65±0.51* (31.6±25.1*)	0.69±0.36* (28.2±14.6*)	0.19±0.20** (17.9±19.7**)	0.90±1.20 (7.7±10.3)	0.20±0.99 (3.2±15.8)

Supplementary Table 3 | NDBC buoy information used in comparison of TC wave duration with ERA5. The missing data percentage is the percentage of missing data duration over the total operating period of buoy. The TC events considered are the TCs, in which Hs ≥2.5m from the buoy when the buoy is within the 15-degree geodesic circle of an ERA5 TC track.

Buoy ID	LON(°)	LAT(°)	Basin	Time period	Missing data Percentage(%)	Sampling interval(hour)	TC number considered
52202	144.8	13.7	WNP	2012.10.25-2020.12.31	38.3	0.5	23
52211	145.7	15.3	WNP	2012.10.25-2021.06.28	33.1	0.5	20
52200	144.8	13.4	WNP	2004.09.08-2021.12.31	7.3	0.5	29
46059	230.0	38.0	EP	1994.10.19-2021.12.31	21.6	1	5
52009	215.3	13.7	EP	1990.09.17-1994.04.11	35.9	1	2
51004	207.6	17.5	EP	1984.11.08-2021.12.21	15.4	1	59
51100	206.1	23.6	EP	2009.04.23-2015.04.28	25.1	1	3
51000	206.2	23.5	EP	2009.04.23-2020.12.31	10.0	1	15
42002	266.3	25.8	NA	1973.06.22-2021.12.31	16.1	1	45
42001	270.3	25.9	NA	1975.08.13-2021.07.25	17.9	1	45
42054	272.3	26.0	NA	2000.07.01-2001.05.14	39.0	1	1
42003	274.4	26.0	NA	1976.11.20-2021.12.01	15.8	1	39
41060	309.1	14.8	NA	2011.11.30-2018.08.28	20.5	1	2
41040	307.0	14.5	NA	2005.05.30-2021.12.31	9.5	1	25
41044	301.3	21.7	NA	2009.05.12-2021.12.31	1.6	1	31
41049	297.0	27.5	NA	2009.05.14-2021.12.31	0.9	1	27
42060	296.5	16.5	NA	2009.04.28-2021.12.31	18.4	1	11
41048	290.4	32.0	NA	2007.09.18-2021.12.31	6.4	1	34
44004	289.6	38.5	NA	1977.09.21-2008.03.08	19.0	1	47
41046	289.1	23.8	NA	2007.09.22-2021.12.31	4.7	1	28
44066	287.4	39.6	NA	2009.06.19-2021.12.31	30.2	1	16
41001	287.3	34.7	NA	1976.06.01-2021.09.14	34.3	1	75

Supplementary Table 4 | Linear trends (after removing the ENSO effect) in intensity (maximum sustained wind) and TC wave height, TC wave area, TC wave accumulated duration and TC wave energy, at global and basin-wide scales, based on two types of TC datasets from ERA5 over 1979–2022. Relative trends are also provided, which are the absolute trend values relative to the 44-year mean. The ERA5 tracks are matched and truncated to IBTrACS (T1). * indicates a significant trend at the 95% confidence level. ** indicates a significant trend at the 90% confidence level. Trends without * or ** do not pass the significance test at the 90% level.

			Global	NH	SH	WNP	EP	NA	NI	SI	SP
TC tracks (T1) (1979 – 2018)	TC intensity	6-hourly-Max wind speed m/s /decade(%)	0.43±0.11* (2.5±0.6*)	0.44±0.13* (2.6±0.7*)	0.46±0.19* (2.7±1.1*)	0.36±0.21* (3.9±1.1*)	0.39±0.37* (2.3±1.4*)	0.39±0.38* (2.2±2.1*)	0.59±0.40* (3.6±2.5*)	0.50±0.22* (3.0±1.3*)	0.48±0.31* (2.7±1.8*)
	TC wave height	6-hourly-Max Hs m/decade(%)	0.18±0.05* (3.7±1.0*)	0.17±0.06* (3.5±1.3*)	0.22±0.08* (4.7±1.8*)	0.26±0.08* (5.0±1.6*)	0.17±0.10* (4.2±2.6*)	0.22±0.20* (4.6±4.1*)	0.16±0.17* (3.9±4.0*)	0.23±0.10* (5.2±2.2*)	0.19±0.17* (3.9±3.4*)
	TC wave area	≥2.5m 10^5km^2 /decade(%)	1.10±0.51* (6.9±3.2*)	0.96±0.47* (7.8±3.9*)	2.06±0.89* (9.2±3.6*)	1.32±0.64* (9.1±4.4*)	1.44±0.72* (16.7±8.4*)	1.64±0.93* (17.9±10.2*)	0.13±1.56 (1.2±14.2)	1.82±0.97* (8.1±4.3*)	1.08±1.55 (4.8±6.9)
	TC wave duration	Mixed wave day/decade(%)	16.0±25.6* (2.9±4.6*)	18.9±12.8* (5.4±3.7*)	-2.9±17.1 (-1.4±5.5)	-12.4±10.4* (-6.5±5.5*)	16.3±8.2* (23.5±11.9*)	9.9±6.8* (14.2±9.8*)	3.1±2.0* (15.5±10.4*)	0.14±11.8 (0.1±8.6)	-3.1±8.3 (-4.6±12.4)
	TC wave energy	Mixed wave 10^{15}kJ /decade(%)	2.73±1.72* (10.6±6.6*)	1.69±0.91* (12.5±6.7*)	1.04±1.11** (8.5±9.0**)	0.42±0.81 (4.6±8.7)	0.53±0.28* (35.1±18.4*)	0.63±0.29* (29.6±13.7*)	0.10±0.11** (16.9±18.2**)	0.93±0.76* (11.7±9.6*)	0.08±0.68 (1.9±15.4)
